# A QbD-based method for the simultaneous determination of tadalafil, terazosin, and tamsulosin using organic-solvent free mixed-micellar HPLC: a sustainable green approach

**DOI:** 10.1186/s13065-026-01866-2

**Published:** 2026-07-01

**Authors:** Yahya Bin Abdullah Alrashdi, Galal Magdy, Mohamed M. Osman, Samy G. Alamir, Sami El Deeb, Ahmed Al-Harrasi, Adel Ehab Ibrahim

**Affiliations:** 1 College of Health Sciences, University of Nizwa, Nizwa, 616 Oman; 2 Pharmaceutical Analytical Chemistry Department, Faculty of Pharmacy, Kafr-Elsheikh University, Kafr Elsheikh, 33511 Egypt; 3 Department of Pharmaceutical Analytical Chemistry, Faculty of Pharmacy, Mansoura National University, Gamasa, 7731168 Egypt; 4 Natural and Medical Sciences Research Center, University of Nizwa, Nizwa, 616 Oman; 5 Department of Pharmaceutical Analytical Chemistry, Faculty of Pharmacy, Mansoura University, Mansoura, 35516 Egypt; 6 Pharmaceutical Analytical Chemistry Department, Faculty of Pharmacy, Ain Shams University, Organization of African Unity Street, Abassia, Cairo, 11566 Egypt; 7 Institute of Medicinal and Pharmaceutical Chemistry, Technische Universitaet Braunschweig, 38106 Brunswick, Germany; 8 Department of Pharmaceutical Analytical Chemistry, Faculty of Pharmacy, Port-Said University, Port Said, 42511 Egypt

**Keywords:** Green analysis, Mixed-micellar liquid chromatography, QbD, Tadalafil, Tamsulosin, Terazosin

## Abstract

The recent updates to benign prostatic hyperplasia treatment from American (2023) and European (2024) guideline recommend the use of alpha-1 blockers in conjunction with phosphodiesterase-5 inhibitors. A new green mixed micellar liquid chromatographic method was established to concurrently analyze alpha-1 blockers, terazosin/tamsulosin, and tadalafil, a major phosphodiesterase-5 inhibitor. Quality by Design was integrated to enhance essential chromatographic variables and resulted in elevated desirability functions. A comprehensive design space was established, guaranteeing the method's reliability. The separation was carried out on a reversed-phase C18 column (150 × 4.6 mm, 5 µm) using organic-solvent-free mobile phase consisting of a mixture of Brij-35 (20 mM), sodium dodecyl sulfate (150 mM), and sodium dihydrogen phosphate buffer (10 mM) adjusted to pH 4.84 and flow rate of 1 mL/min. Ultraviolet detection was performed at 285 nm for tadalafil and 214 nm for terazosin and tamsulosin. The linearity range observed for all drugs under investigation was (5–100 µg/mL). The limits of detection were 0.27, 0.29, and 0.32 µg/mL, and the limits of quantification were 0.81, 0.88, and 0.97 µg/mL for terazosin, tadalafil, and tamsulosin, respectively. Validation in accordance with International Council for Harmonisation guidelines was successfully implemented. The approach was effectively utilized to identify analytes in the formulated tablets and pharmaceutical products. The ecological impact of the method was evaluated using the complex green analytical procedure index and Analytical GREEnness metrics, demonstrating the advantages of the proposed procedure over previously reported methods.

**Supplementary Information:**

The online version contains supplementary material available at 10.1186/s13065-026-01866-2.

## Introduction

Benign prostatic hyperplasia (BPH) is a common, nonmalignant enlargement of the prostate gland that occurs more frequently with age, primarily due to hormonal alterations and altered androgen metabolism [1]. Histologically, BPH is characterized by hyperplastic proliferation in the prostate transition zone, involving both glandular epithelial cells and stromal elements, including smooth muscle and fibrous tissue. The enlargement of the prostate can constrict the urethra and impede urinary flow, resulting in various Lower Urinary Tract Symptoms (LUTs), including urinary frequency, urgency, nocturia, diminished urine stream, hesitation, incomplete bladder evacuation, and occasionally urinary retention [2]. Alongside urinary symptoms, numerous individuals with BPH also encounter Erectile Dysfunction (ED), potentially associated with common risk factors including aging, vascular dysfunction, inflammation, metabolic syndrome, and psychological stress because of persistent urine discomfort. LUTs and ED substantially diminish patients' quality of life by impacting sleep, daily activities, mental well-being, and sexual health, rendering BPH a critical clinical condition for efficient long-term therapy [3].

**Fig. 1:**
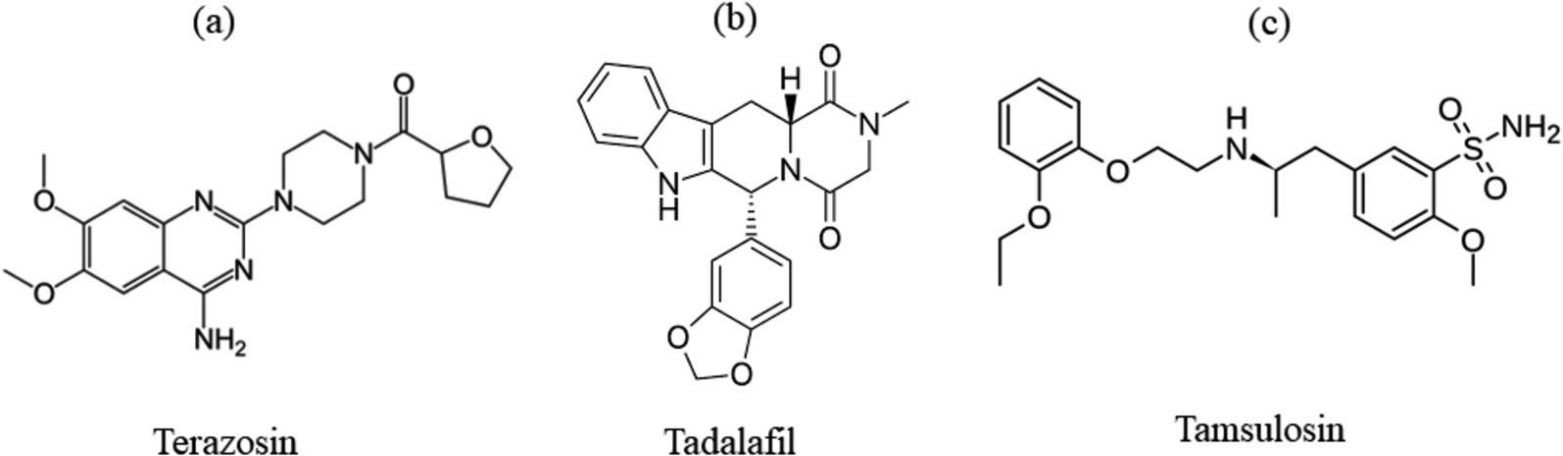
Chemical structure of the selected drugs

According to the American Urological Association (AUA), three classes are used alone or in combination to manage BPH: alpha-blockers, 5α-Reductase inhibitors (5-ARIs), and phosphodiesterase 5 inhibitors (PDE5Is) [4]. Alpha-blockers, including tamsulosin and terazosin (Fig. 1), are often used as first-line treatment, as this approach has been widely studied since the 1980s due to their ability to relax smooth muscle in the prostate and bladder neck, thus improving urinary flow and decreasing LUTS within days of initiation [5]. Despite the comparable efficacy of all alpha-blockers, the choice of medication depends on the patient's medical history and potential side effects. TRZ has been shown to reduce blood pressure, resulting in orthostatic hypotension; hence, care is suggested in individuals already on antihypertensive medication. On the other hand, Tamsulosin (TMS) possesses an increased risk of inducing iris problems and ED [4]. PDE5Is, such as Tadalafil (TAD) (Fig. 1), were used to treat ED and pulmonary arterial hypertension. Later, TAD was approved for the treatment of BPH, as it facilitates vasodilation and relaxes the lower urinary tract and the smooth muscles of the prostate by enhancing the effects of nitric oxide, which inhibits the degradation of cGMP [6]. Concerning the previous, ED could be caused by BPH or a medication side effect. In 2021, the AUA recommended prescribing PDE5Is alone and avoiding low-dose TAD with alpha-blockers for symptomatic management [4]. However, this recommendation was recently changed in the 2023 AUA Guideline Amendment [7] and in April 2024 European Association of Urology guidelines [8, 9]. Clinicians may now offer the low-dose TAD and alpha-blocker combination, although alpha-blocker selection may vary depending on the patient [7]. Consequently, pharmaceutical capsules were formulated containing TAD and TMS that aim to prevent progressive and/or treat BPH and ED with excellent stability and efficacy are now supported [10].

The advancement of environmentally friendly analytical techniques is increasingly important for safeguarding analysts and reducing environmental impacts, particularly in pharmaceutical analysis, where high-performance liquid chromatography (HPLC) is favored for its efficiency, consistency, and ease of use. Nevertheless, conventional reversed-phase HPLC is highly dependent on organic solvents, resulting in considerable hazardous waste generation and posing potential health hazards to laboratory staff. On the other hand, micellar liquid chromatography (MLC), particularly its advanced form, mixed-MLC, effectively addresses these concerns by employing aqueous mobile phases containing both ionic and nonionic surfactants [11]. In contrast to RP-HPLC, which relies on organic solvents for optimal elution, mixed-MLC enables the stationary phase to interact with ionic surfactants via electrostatic retention and with non-ionic surfactants via hydrophobic interactions [12, 13]. This approach effectively mimics the separation efficiency of traditional methods while avoiding the environmental and safety concerns typically associated with them. This environmentally friendly method substantially reduces the waste of toxic solvents while improving safety for analysts by reducing their exposure to hazardous chemicals. Furthermore, implementing mixed-MLC can reduce operational expenses associated with solvent use and disposal and help organizations comply with increasingly stringent environmental regulations [14]. Consequently, unlike conventional RP-HPLC, environmentally friendly analytical approaches that incorporate both ionic and non-ionic surfactants offer a more sustainable, safer, and economically viable option while maintaining high analytical performance [15].

Recently, mixed-MLC methods have been effectively optimized through the application of Quality by Design (QbD) strategies [16]. QbD is a systematic, science-based, and modern approach employed primarily in pharmaceutical development [17], quality assurance [18], and monitoring [19]. It guarantees uniform quality by developing an in-depth understanding of process variables, identifying essential parameters, and maintaining them within clearly defined control limits to achieve the desired quality characteristics. In recent years, QbD principles have been effectively applied beyond pharmaceutical formulation to the development of analytical and bioanalytical procedures, resulting in significant enhancements over conventional trial-and-error methodologies [20]. In contrast to the traditional one factor at a time (OFAT) methodology, QbD offers a more efficient and environmentally friendly approach by reducing experimental waste, lowering solvent use, and minimizing analysis time. Moreover, it facilitates the concurrent assessment of multiple elements and their interactions, thereby enhancing method robustness and ensuring reliable performance despite minor fluctuations in experimental conditions [21]. Response surface methodology (RSM), together with the central composite design (CCD), is widely used to develop and optimize analytical methods within the QbD frameworks. RSM employs statistical and mathematical modeling to elucidate the relationships between key process variables and significant analytical responses, including resolution, retention time, peak symmetry, sensitivity, and robustness [22]. These models assist in predicting method efficacy, determining optimal operational parameters, and developing a design space in which the method reliably satisfies established requirements with minimal predictive error. Consequently, RSM–CCD facilitates the development of reliable, reproducible, high-quality analytical methodologies through fewer experimental trials and improved regulatory compliance [23].

Although multiple analytical techniques were reported to determine the drugs under study, TRZ, TMS, or TAD, individually in different matrices, only few techniques have been reported for their combined formulations analysis. TMS/TAD combination has been analyzed using liquid chromatography [24, 25], thin layer chromatography [26], fluorescence spectroscopy [27, 28] and UV–Vis spectroscopy [29]. TMS and TRZ have been simultaneously determined using liquid chromatography [30, 31] and micellar electrokinetic chromatography [32]. On the other hand, no analytical method has been reported for the simultaneous determination TRZ/TAD combination yet. None of the previously reported analytical methods has been able to simultaneously analyze the three drugs under study in a single run.

This study is aimed at reporting the first green, QbD-driven mixed-micellar liquid chromatographic method capable of simultaneously quantifying TRZ, TMS, and TAD in a single run—addressing an important analytical gap because earlier literature largely focused on individual drugs or partial two-drug combinations, and none achieved concurrent determination of all three agents under environmentally preferable conditions. The method’s novelty lies in its sustainable reversed-phase alternative: a fully organic-solvent-free aqueous mobile phase composed of Brij-35 and SDS was designed to deliver effective chromatographic selectivity through combined hydrophobic and electrostatic interactions, thereby eliminating the hazardous solvent consumption commonly associated with conventional RP-HPLC. Beyond analytical chemistry performance, the procedure was scientifically aimed to be strengthened by a QbD framework implemented through CCD and response modeling to identify critical chromatographic factors, optimize their interactions, and construct a validated design space. This strategy improves robustness and method reliability by defining operational ranges that ensure consistent separation, acceptable peak behavior, and reproducible sensitivity even with minor experimental variations—moving beyond traditional one-factor-at-a-time optimization. Finally, the research adds a sustainability dimension by assessing the method’s environmental impact using ComplexGAPI and AGREE metrics and demonstrating improved greenness relative to previously reported LC approaches. Collectively, the proposed method provides a dual contribution: (i) an efficient analytical workflow for combination BPH/ED therapies and (ii) a greener, QbD-validated platform suitable for routine quality control and regulatory-aligned method performance.

## Experimental

### Chemicals and materials

All reagents utilized were of analytical grade. The source of Brij-35 was Sigma-Aldrich (Massachusetts, USA). Methanol, sodium hydroxide, sodium dihydrogen phosphate, phosphoric acid, and sodium dodecyl sulfate (SDS) were obtained from Merck (Darmstadt, Germany). Nylon membrane filters (0.45 μm) were purchased from Teknokroma (Barcelona, Spain). Stuart A4000 Aquatron water still (New Jersey, USA) was used to supply fresh deionized water. TMS, TRZ and TAD pure standards were kindly supplied by the Egyptian International Pharmaceutical Industries Co. (EIPICo, Tenth of Ramdan city, Egypt). Marketed formulation of Urimax-T® Capsule produced by Cipla Ltd. (Mumbai, India) was purchased from the Indian market. Itrin® tablets, produced by Abott (TRZ, 2 mg per tablet), and Starkoprex® tablets, produced by Multi-Apex Pharma (TAD, 5 mg per tablet) were purchased from the Egyptian market.

### Instrumentation

All analytical chromatographic runs for the study were carried out with the aid of a Shimadzu HPLC device, Model: Nexera-i LC-2040C 3D Plus (Tokyo, Japan). The system has the components of an auto-sampler system, column compartment, and photodiode array detector. A Kinetex RP-C18 column was used for chromatographic separation (150 × 4.6 mm, 5 μm), which was manufactured by Phenomenex (California, USA). pH adjustment was performed using SevenDirect SD20 pH-meter produced by Mettler-Toledo (Greifensee, Switzerland).

### Stock solutions, working dilutions, and quality control standards

Primary stock solutions of each drug under investigation were prepared at concentration of 1.0 mg/mL in ethanol. Mixed dilutions were then prepared from these stock solutions using the mobile phase. The standards were preserved in refrigerator between 4 and 8 °C. The validation process was successfully done through serial concentrations of mixed drugs’ standards.

### Procedure for prepared tablets and dosage form

The content of one Urimax-T® Capsule, composed of TMS/TAD (0.4/5.0 mg per capsule), was placed in 50 mL volumetric flask, mixed carefully with 10 mL mobile phase, vortex-mixed, then brought to volume using the mobile phase. Laboratory prepared mixture for the TRZ/TAD combination was prepared by mixing the content of 1 tablet of Itrin® (2 mg TRZ) and Starkoprex® (5 mg TAD), after being crushed, and then thoroughly mixed with 10 mL mobile phase. The solution was then prepared to a volume of 100 mL.

### Study design

#### Central composite design (CCD)

To effectively study the separation of the target analytes, 19 distinct mobile phase compositions were prepared and used in the chromatographic analysis (Supplementary materials Additional file 1: Table S1). The goal was to determine how different Brij-35 and SDS concentration ratios, as well as the mobile phase's pH, affected the separation. Design-Expert® software (version 11, Stat-Ease Inc., Minneapolis, USA) was employed to develop experimental design, perform factor screening and chromatographic optimization. The CCD variables—pH and the concentrations of Brij-35 and SDS—were selected because preliminary experiments showed that these factors most strongly influenced key chromatographic responses (notably retention time and resolution). The selected ranges of the CCD factors were determined based on preliminary chromatographic trials. The pH range of 3.5–5.5 was selected to test optimal analyte ionization, satisfactory peak shape, and peaks’ resolution. The concentration ranges of Brij-35 (16.0–35.0 mM) and SDS (70.0–130.0 mM) were chosen to test their ratios capability for fast elution and enhanced resolution between drugs’ peaks (Additional file 1: Table S1). Conditions beyond these ranges resulted in inadequate separation, excessive retention, or distorted peak shapes. Depending on the required concentrations, particular weights of Brij-35 and SDS were dissolved in 800.0 mL of deionized water to establish the mobile phase. After adding 10.0 mM of monobasic sodium phosphate, the pH was adjusted with either 1.0 M sodium hydroxide or 1.0 M phosphoric acid. Then, de-ionized water was used to bring the final volume to 1000 mL. The mobile phase solution was filtered using 0.45 μm nylon Millipore filters. The column was conditioned with the mobile phase for 15 min at a flow rate of 1.0 mL/min prior to sample injection. For analysis, a 20 μL sample volume was injected. TAD was detected at 285 nm, while TRZ and TMS were detected at 214 nm. Prior to conditioning with the subsequent mobile phase, adsorbed surfactants were eliminated from the stationary phase following each run by flushing the system with a 50:50 (v/v) ethanol–water mixture for 15 min.

#### Analysis response and optimization

Four analytical responses were assessed, including the retention times of TRZ and TMS (tR_TRZ_ and tR_TMS_) and the chromatographic resolution between neighboring peaks, namely (Rs_TRZ/TAD_ and Rs_TAD/TMS_). An appropriate statistical model has been developed for each response to describe its relationship with the important technique variables. The model's suitability was evaluated using ANOVA by examining *p* values, lack-of-fit tests, significant model terms, adjusted and anticipated R^2^ values, and appropriate precision.

The optimization step was performed numerically and graphically by imposing constraints to minimize tR_TRZ_ and tR_TMS_ and to target analyte-to-analyte Rs of at least 2. The confidence interval function provided additional information about the precision of the analysis.

### Method validation

The system suitability, Specificity, Selectivity, Linearity, calibration curve, Range, Accuracy, Precision, limits of detection and quantification (LOD and LOQ), and robustness of the developed method were evaluated in accordance with ICH recommendations [33].

#### System suitability and selectivity

Prior to analysis, system suitability was evaluated to confirm efficient chromatographic performance. The evaluation involved parameters such as retention time, peak area repeatability, theoretical plate count, tailing factor, and resolution between critical peak pairs, utilizing replicate injections of the mixed standard solution at mid-range (50 µg/mL). The system was deemed appropriate once all values satisfied the established acceptance criteria. An overlay spectrum of the 3 drugs has been studied (Additional file 1: Fig. S2). The selected wavelengths were chosen based on the UV absorption spectra of the studied drugs to achieve maximum sensitivity for detection and/or appropriate selectivity.

The selectivity of the proposed method was confirmed by evaluating its effectiveness in resolving TRZ, TAD, and TMS in a mixed standard, ensuring no peak overlap. Furthermore, the method's specificity was demonstrated by its ability to distinctly separate the analytes in the presence of the mobile-phase blank and excipient/background components, with no observed interference.

#### Linearity and calibration curve

The investigation of linearity involved analyzing a set of calibration standards prepared at various concentrations spanning the estimated working range. Calibration curves were developed to determine the slope, intercept, and correlation coefficient. For the linearity, 7 concentrations were prepared to achieve a fruitful linear relationship. Standard concentrations (5.0, 10.0, 20.0, 40.0, 60.0, 80.0 and 100.0 µg/mL) were the exact concentrations to achieve the required calibration curve for all drugs of the study. The method appeared linear, as evidenced by satisfactory regression statistics and minimal deviation in back-calculated concentrations.

The sensitivity of the method was assessed by estimating the limits of detection (LOD) and quantification (LOQ) through formulas LOD = 3.3σ/S and LOQ = 10σ/S, where σ is the standard deviation of the response, and S is the slope of the calibration curve [33, 34].

#### Accuracy and precision

The method’s accuracy was evaluated through recovery experiments, in which samples were spiked with known quantities of the analytes at low, medium, and high concentrations. The drugs’ quality control samples were spiked in a placebo solution containing some commonly co-formulated excipients (Magnesium stearate, starch, avicel-101, cross carmellose sodium, and titanium dioxide) at concentrations 0.5 mg/mL in the mobile phase. Three concentrations were reliable to fulfill the required validation parameters and align with quality control aspects. The first concentration (LQC) was 10.0 µg/mL, the second concentration (MQC) was 40.0 µg/mL, and finally the third one (HQC) was 80.0 µg/mL. These concentrations were assigned for all drugs. The calculation of %recovery involved comparing the measured concentration with the nominal value.

Precision was assessed using repeatability (intra-day) and intermediate precision (inter-day) by analyzing quality-control samples at three concentration levels. The repeatability evaluation was conducted on the same day, whereas the intermediate precision was determined across three different days. Precision was expressed as the %RSD of the measured concentrations.

#### Robustness

The examination of robustness involved introducing minor, deliberate variations in key chromatographic conditions, including flow rate, temperature, and detection wavelength. The impact of these changes on the %recovery was demonstrated. The method was considered robust when no notable loss of %recovery was detected. It’s important to note that the robustness was augmented by the QbD study as shall be discussed.

## Results and discussion

### Response surface methodology and method development

#### Central composite design (CCD)

Central composite design (CCD) is one of the most commonly utilized experimental designs to generate second-order (quadratic) models that illustrate the relationship between analytical responses and key method variables. This approach allows for a structured assessment of both the individual and collective impacts of various factors on one or more outcomes, and it can be adapted to accommodate numerous responses within the same experimental environment. The overall count of experimental runs in a CCD is dictated by the number of variables under investigation (k) and consists of three distinct sets of points: factorial runs (2^k), axial (star) runs (2k), and replicated center runs. This study selected a rotatable CCD due to its ability to deliver uniform prediction variance at equal distances from the design center, thereby enhancing stability in comparison to non-rotatable designs. The axial distance (α) is dependent on the number of factors and is calculated according to the relationship α = (2^k)^(1/4) [35]. In this context, each variable was analyzed at five coded levels (− α, − 1, 0, + 1, + α), with ± α indicating the axial points, ± 1 signifying the low and high factorial levels, and 0 representing the center point. Herein, CCD was utilized within the Response Surface Methodology framework to assess the impact of pH (A), Brij concentration (B), and SDS concentration (C) on the chromatographic performance of the proposed method. The coded and actual levels of the investigated factors are summarized in Additional file 1: Table S3. The design facilitated the simultaneous investigation of how these variables influenced the retention times of the analytes (tR of TRZ, TAD, and TMS) and the required resolutions between neighboring peaks (Rs_(TRZ/TAD)_ and Rs_(TAD/TMS)_). The factorial points facilitated the estimation of the main and interaction effects among the variables under investigation, while the axial points enabled the identification of curvature and the modeling of quadratic behavior in both retention and resolution.

#### Optimization of analysis response

The responses analyzed were subjected to statistical analysis to identify factors that significantly affect chromatographic performance. Table 1 presents an outline of the polynomial regression equations developed for each response, detailing the primary effects of the variables, their interactions, and their quadratic contributions. Appropriate response transformations were applied as needed to improve model fit. ANOVA results indicated that all proposed models were highly significant (*p* < 0.0001), whereas lack-of-fit tests were not significant (*p* > 0.05), suggesting a strong alignment between the experimental and predicted values. The refinement of the model by retaining only statistically significant terms improved predictive reliability, as evidenced by the close alignment between the adjusted and predicted R^2^ values. Furthermore, adequate precision values (> 4) indicated a satisfactory signal-to-noise ratio, supporting the suitability of the models for navigation within the design space [36]. The impact of each factor on a specific response can be determined from the magnitude and sign of its regression coefficient, with larger coefficients indicating greater effects. In this study, pH had a negligible effect on tR_TRZ_ and tR_TAD_, and no significant effect on Rs_TRZ/TAD_ or Rs_TAD/TMS_. In contrast, Brij and SDS concentrations emerged as the primary factors influencing both retention behavior and peak resolution.

**Table 1:** Statistical parameters of the models for selected responses

Response models*	ANOVA		Adjusted R^2^	Predicted R^2^	Adequate precision
	*p* value	Lack of fit			
1/Sqrt(tR_(TRZ)_) = 0.3985 + 0.0004 A + 0.0017 B + 0.0460 C -0.0038 A^2^ + 0.0082 B^2^ − 0.0031 C^2^	< 0.0001	0.7553	0.9972	0.9962	112.9069
1/(tR_(TAD)_) = 0.1366 − 0.0007 A + 0.0142 B + 0.0210 C + 0.0017 BC − 0.0020 A^2^	< 0.0001	0.2164	0.9898	0.9810	58.1894
1/(tR_(TMS)_) = 0.0929 + 0.0042 B + 0.0217 C + 0.0017 BC	< 0.0001	0.1085	0.9966	0.9951	140.4037
Rs_(TRZ/TAD)_ = 1.39 − 0.8182 B + 0.4757 C	< 0.0001	0.1242	0.9702	0.9550	47.3476
(Rs_(TAD/TMS)_)^2.6 = 45.87 + 15.48 B − 27.76 C − 5.38 BC − 5.96 B^2^ + 10.24 C^2^	< 0.0001	0.1012	0.9973	0.9931	123.0654

^*^Significant factors: 1/Sqrt(tR_(TRZ)_) = inverse square root of terazosin retention time; 1/(tR_(TAD)_) = inverse of tadalafil retention time; 1/(tR_(TMS)_) = inverse of tamsulosin retention time; Rs_(TRZ/TAD)_ = resolution between terazosin and tadalafil; (Rs_(TAD/TMS)_)^2.6 = power transformation of resolution between tadalafil and tamsulosin; A = pH; B = Brij concentration; C = SDS concentration

**Fig. 2:**
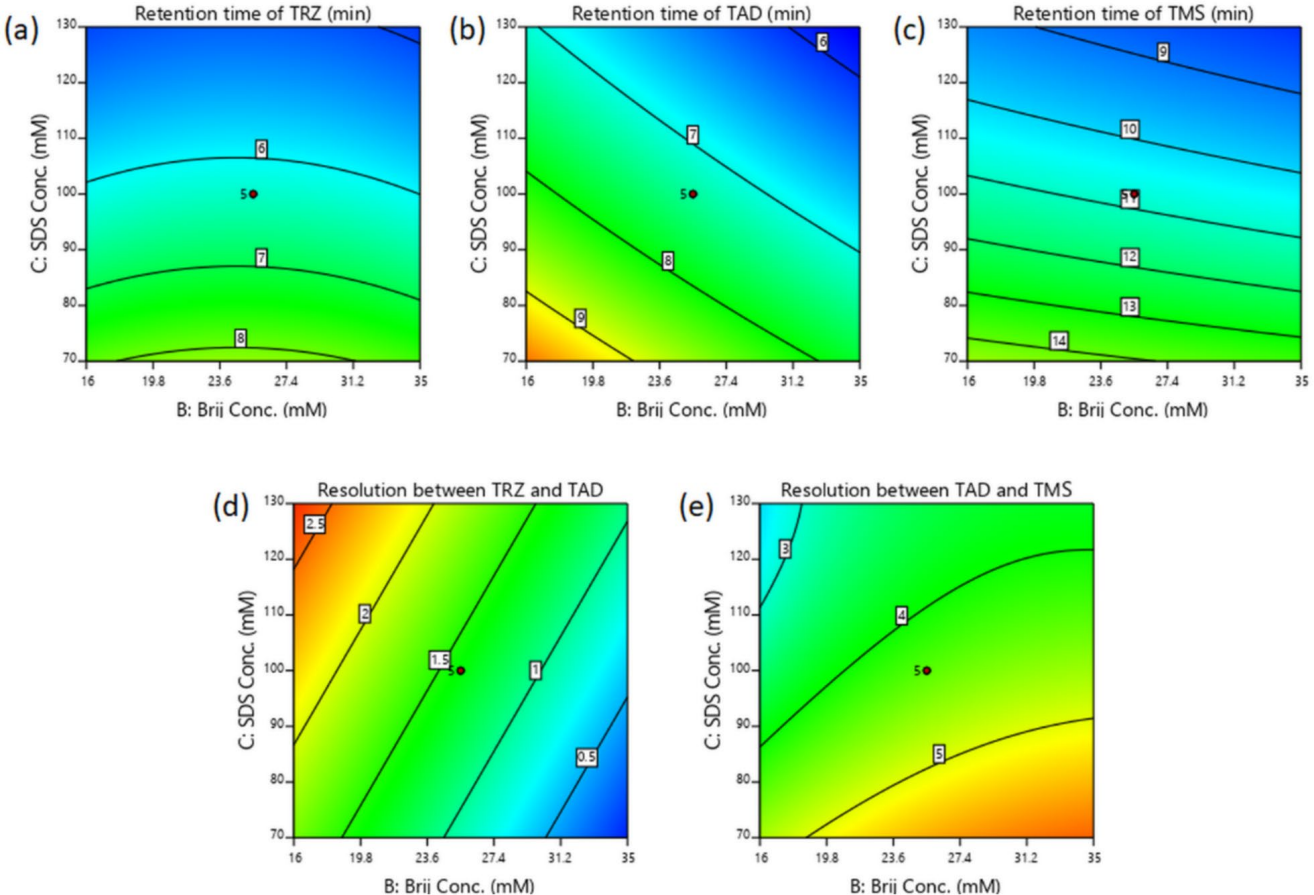
Contour plot for the effect of significant variables on the selected responses

**Fig. 3:**
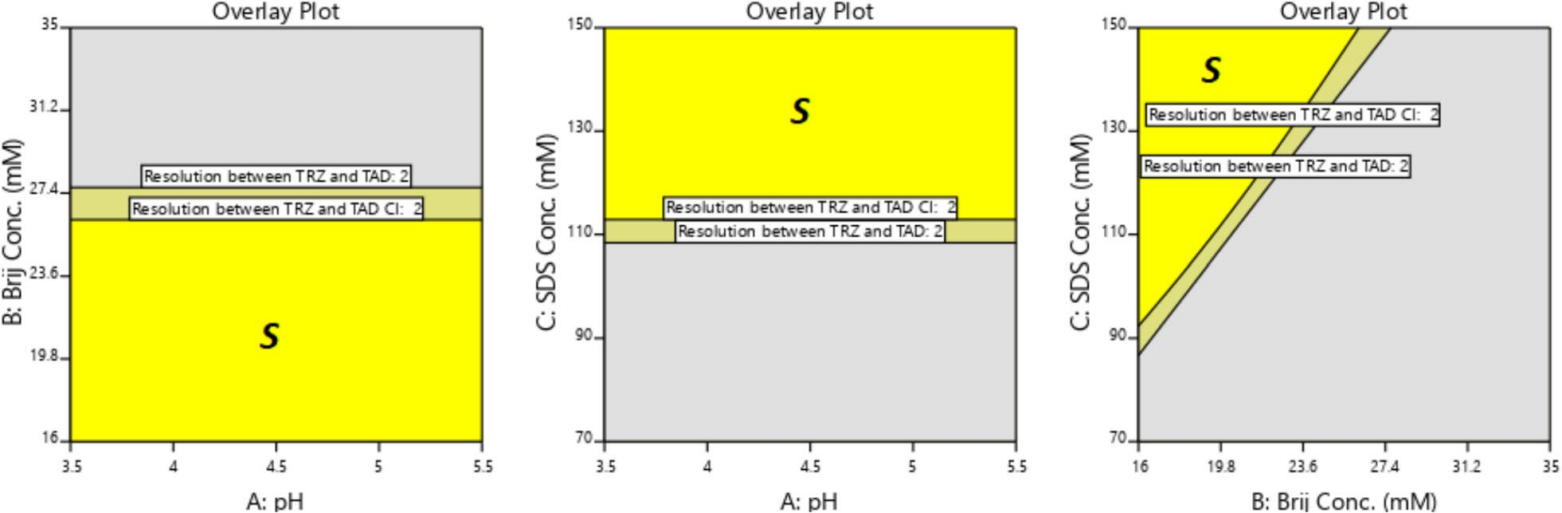
Overlay Plot for the selected responses with the sweet spot (***S***)

Based on the contour plots (Fig. 2) of the selected responses against the combination of the most significant factors, Brij concentration (factor B) showed a negative effect on tR_TAD_, tR_TMS_, and Rs_(TRZ/TAD)_ (Fig. 2b–d) but a high positive effect on Rs_(TAD/TMS)_ (Fig. 2e), and a quadratic one on tR_TRZ_, (Fig. 2a). On the other hand, SDS concentration had a positive effect on Rs_(TRZ/TAD)_ (Fig. 2d) while negatively affecting the remaining responses (Fig. 2a–c, e). After setting constraints on variables and responses, the optimization step was carried out using numerical, graphical, and Derringer and Suich desirability functions. The numerical optimization suggested condition, with a desirability of 0.641, was a pH of 4.84, 20 mM Brij, and 150 mM SDS. Graphical optimization specifies the design space (robustness zone) via an overlay plot that represents all targeted response contours on a single contour. The overlay plots were generated using desirability optimization to identify the experimental region that simultaneously met the predefined chromatographic criteria. The sweet spot (bright yellow region, S) represents the optimum operating space providing adequate resolution and satisfactory chromatographic performance for the studied drugs in addition it demonstrates a robust zone as small variations in pH, Brij-35 concentration, and SDS concentration within this experimental space did not significantly affect chromatographic performance; the area shaded in gray does not meet the optimization criteria; and the dark gold indicates a doubtful response estimate. The optimum variable ranges of the overlay plots were pH (3.5–5.5), Brij concentration (16.0–35.0), and SDS concentration (93.3–150.0 mM) (Fig. 3). The post-analysis point prediction confirmed that the mean of all responses fell within the 95% prediction interval (PI low 95% and PI high 95%).

### Mechanistic role of SDS and Brij-35 in the mixed micellar chromatographic system

The mixed micellar system composed of SDS and Brij-35 exerted a synergistic influence on the chromatographic behavior of the substances analyzed. SDS, an anionic surfactant, forms negatively charged micelles that improved analyte partitioning into the micellar phase and diminished analyte interactions with the stationary phase, thereby altering retention behavior and chromatographic selectivity [37]. Increasing SDS concentration enhances micellar solubilization of the analytes, thereby reducing retention times. In contrast, Brij-35, a nonionic surfactant, affects the hydrophobic microenvironment of the mixed micelles and altered analyte-micelle interactions, leading to increased selectivity and enhanced chromatographic resolution, especially between closely eluting peaks [37]. The combined use of SDS and Brij-35 facilitated synergistic electrostatic and hydrophobic interactions [13, 38], enhancing the simultaneous separation efficiency of TRZ, TAD, and TMS in the proposed mixed micellar chromatographic system.

### Validation of the HPLC method

#### System suitability

System suitability testing was conducted to ensure efficient chromatographic performance prior to the validation and routine application of the proposed method. The developed separation yielded well-resolved, symmetrical peaks for TRZ, TAD, and TMS, showing short retention times, valued theoretical plate counts, and peak separation. The resolution values exceeded 2.0, confirming baseline separation of the analytes, and peak symmetry was satisfactory for all analytes (Table 2). The results collectively confirmed that the chromatographic system can successfully achieves satisfactory fast analysis in short retention times, effective base-line separation, and reliable quantification of the analyzed drugs.

**Table 2:** Linearity results for the determination of the drugs under study using the proposed conditions

Parameter	TRZ	TAD	TMS
Retention time (min. ± RSD)	4.27 ± 0.41	5.78 ± 0.45	8.33 ± 0.34
Theoretical plates, N*	1375	1741	2335
Resolution	–	4.33	4.16
Symmetry factor	1.09	1.15	1.06
Linearity range	5.00–100.00 µg/mL		
Detection wavelength	214 nm	285 nm	214 nm
Linearity equation	y = 1200.3x + 20500	y = 83116x + 6129.6	y = 45128x − 17050
Determination coefficient (R^2^)	0.9997	0.9999	0.9999
LOD (µg/mL)	0.27	0.29	0.32
LOQ (µg/mL)	0.81	0.89	0.97

^*^N calculated per 15 cm column length

#### Selectivity and specificity

**Fig. 4:**
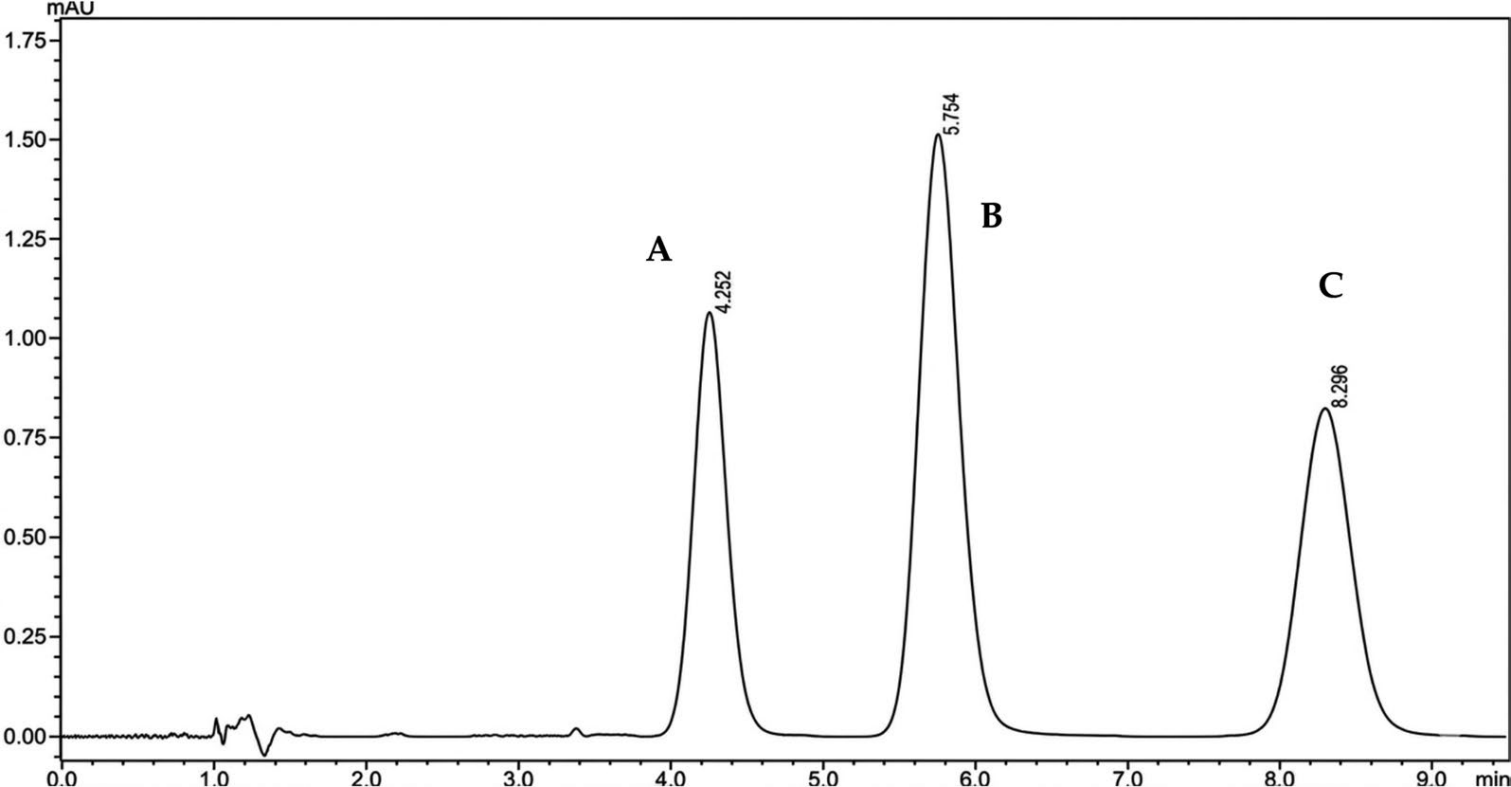
Chromatogram showing separation between TRZ (**A**), TAD (**B**) and TMS (**C**) using the proposed green organic-solvent free optimized chromatographic conditions

**Fig. 5:**
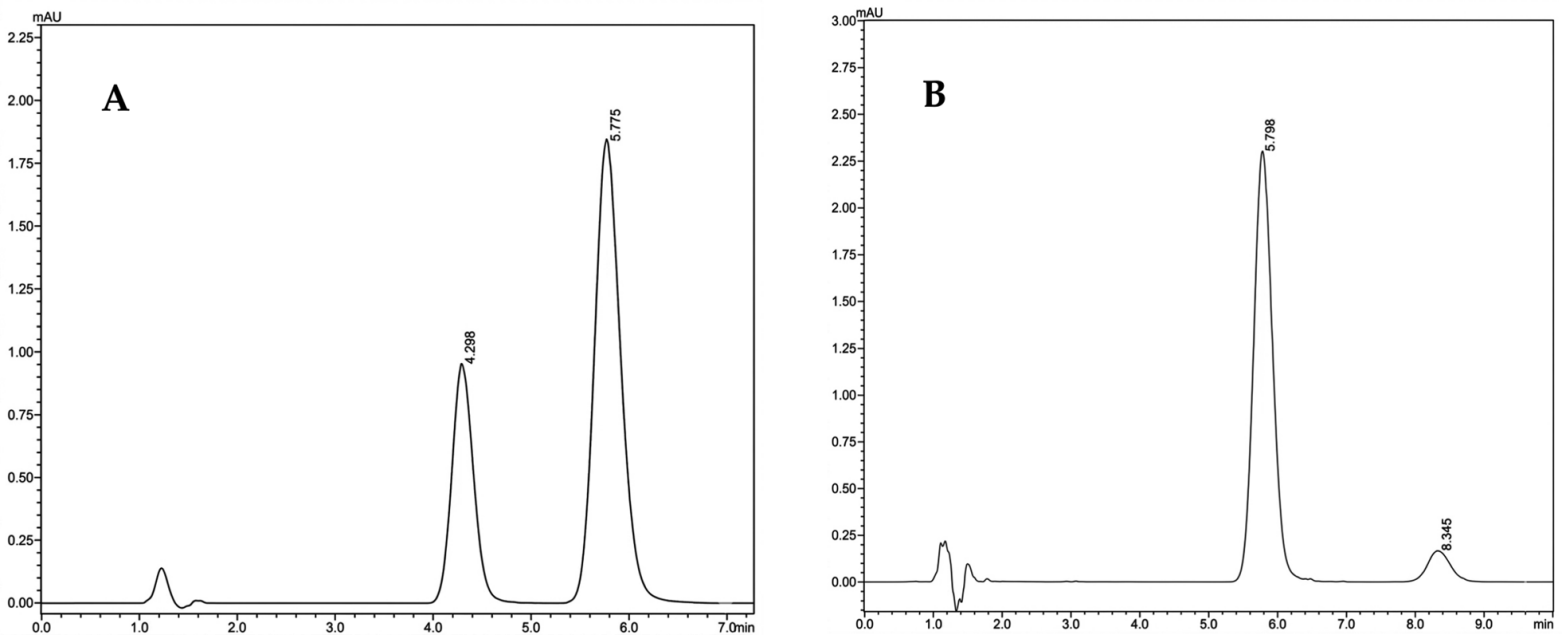
Determination of TRZ/TAD in Laboratory prepared mixture (**A**), and TAD/TMS in Urimax-T® capsule (**B**) using the mixed-MLC optimized conditions

The proposed mixed-micellar HPLC method exhibited notable selectivity, as evidenced by the distinct chromatographic separation of TRZ, TAD, and TMS within the mixed standard solution. Figure 4 shows that the three analytes were completely resolved under the selected optimum chromatographic conditions, with no peak overlap, thereby validating the method's ability to differentiate each compound from the others. The observed chromatographic behavior demonstrates suitable selectivity of the optimized micellar system, facilitating dependable simultaneous determination of the analyzed drugs. Furthermore, method specificity was evaluated by the absence of interferences from excipients with the drugs under study in their pharmaceutical dosage forms (Fig. 5). As shown (Fig. 5), each drug generated a distinct, sharp, and clearly defined peak at its specific retention time, with no signs of overlapping signals, thereby validating the specificity of the method response for each analyte. The results demonstrate that the established method is specific and appropriate for routine assessment of the target analytes in both bulk and dosage form samples.

#### Linearity and calibration curve

The linearity of the proposed chromatographic method was assessed by constructing calibration curves for TRZ, TAD, and TMS over the concentration range 5.0–100.0 μg/mL. Solid linear relationships were established between peak area and analyte concentration throughout the examined ranges. The regression equations are as follows: y = 1200.3x + 20500 for TRZ, y = 8311.6x + 6129.6 for TAD, and y = 45128x − 17050 for TMS, where y was the detector response and x was the corresponding analyte concentration. The calibration curves demonstrated exceptional correlation coefficients, with determination coefficients (R^2^) of 0.9997, 0.9999, and 0.9999 for TRZ, TAD, and TMS, respectively, validating the method's efficacy for analyte quantification (Table 2).

Based on the data presented in Table 2, the sensitivity of the developed method was clearly demonstrated by its calculated LOD and LOQ values as proposed by the ICH guidelines [33].

#### Accuracy and precision (repeatability and intermediate precision)

For measuring the degree of proximity between the true linear concentrations and the tested sample values, triplicate injections of each concentration of the QC levels were assigned for this purpose. The accuracy results showed distinct recovery percentages with small relative standard deviations, as shown in Table 3, indicating the closeness of the calculated results to their true values.

**Table 3:** Accuracy and precision results for the determination of the drugs under study using the developed method

Drug	TRZ		TAD		TMS	
	R%	RSD	R%	RSD	R%*	RSD**
Standard (µg/mL)	Repeatability					
10	101.56	1.24	98.08	0.55	101.38	0.93
40	100.74	0.97	98.91	0.92	100.64	2.05
80	100.26	0.09	101.42	0.59	99.72	0.68
	Intermediate precision					
10	102.07	1.89	98.04	0.02	101.18	0.82
40	100.37	0.32	98.29	0.59	101.03	0.78
80	100.49	0.16	100.49	0.08	99.94	0.34
	Accuracy					
10	101.37	2.82	99.09	0.53	101.44	0.32
40	100.01	0.96	98.92	0.48	99.55	0.07
80	100.55	0.41	101.54	0.45	100.59	0.86

^*^Recovery% = calculated concentration/Actual concentration × 10

^**^RSD relative standard deviation

Table 3 demonstrates good precision values of the tested QC samples on both levels of precision. The intra-day testing offered excellent recovery results, which referred to the achievement of precise repeatability. On the three successive days of measurements (Inter-day precision), the three quality control concentrations displayed comparable R% within low deviations within acceptable range, which indicated overall good precision.

#### Robustness

Slight changes in the optimized chromatographic conditions have to be made, according to ICH guidelines, to confirm the robustness of the resulting method. Different parameters were monitored, such as flow rate (tuned by ± 0.1 mL/min), run temperature (tuned by ± 2 °C), and wavelength (tuned by ± 2 nm). All calculated results in comparison with their original responses were acceptable within low relative standard deviation (RSD) values (Additional file 1: Table S4) and manifested high robustness of the method against any alterations that might occur. It should also be noted that the chromatographic parameters were first selected within the robust sweet spot zone during the QbD study. This in turn supports the hypothesis.

### Application to pharmaceutical dosage forms assay

Applying newly developed analytical methods for pharmaceutical determination in their dosage forms is crucial to ensure efficacy in quality control. It provides more accurate, sensitive, and good evidence on their validity for use the intended purpose in routine measurement of active ingredients throughout development and production. That in turn enables faster batch release, better detection of degradation and instability, improving compliance with increasingly stringent regulatory standards, and ultimately protecting patient health and supporting innovation in formulation and manufacturing.

On a commercial quality control level, the developed method was applied for the determination of the drugs under study in their pharmaceutically marketed dosage forms; TRZ (2 mg/tablet), TMS/TAD (0.4/5.0 mg per capsule), and TAD (5 mg/tablet). These dosage forms strengths were selected because they are commonly marketed and have demonstrated therapeutic efficacy in managing BPH and its related symptoms [39, 40]. In addition, as mentioned earlier, guidelines have recommended the use of combination of alpha-blockers and PDE5Is to manage BPH [4]. TMS/TAD combinations proved better improvements on the LUTs associated with BPH [41]. Therefore, this combination was formulated and marketed by a considerable number of pharmaceutical companies. Urimax-T® Capsule was analyzed for their labeled TMS/TAD content (Table 4, Fig. 5). The results obtained confirm the agreement between the labeled and determined contents. Although no marketed dosage forms were available for the TAD/TRZ combination, a laboratory prepared mixture was applied by mixing the contents of TAD (5 mg Starkoprex® tablets) and TRZ (2mg Itrin® tablets) tablets, to simulate a proposed formulation and challenge the newly developed method for their combined determination. The results (Table 4, Fig. 5) indicate the capability of the method to be utilized in their quality control operations.

**Table 4:** The results of the application of the proposed method on pharmaceutical dosage forms

Dosage forms (Company)	Ingredients	Dose per tablet (mg)	Assay%* ± RSD
Urimax-T® Capsule (Cipla, India)	TAD	5.00	100.37 ± 0.99
	TMS	0.4	98.29 ± 1.92
Laboratory prepared mixture	TAD	5.00	100.42 ± 0.41
	TRZ	2.00	100.48 ± 0.69

^*^Average assay percentages and relative standard deviations (*n* = 3)

### Ecological assessment of the proposed method

Assessment of the ecological footprint of newly developed analytical methodologies became one of the basic steps in their evaluation [42]. The ecological effect of the developed mixed-micellar HPLC method was fully evaluated using two complementary tools, ComplexGAPI [43] and AGREE [44], to guarantee a comprehensive and reliable assessment of sustainability. ComplexGAPI was utilized to explore the complete analytical workflow, including sample preparation, reagent types and hazards, energy consumption, and waste production [43]. The ComplexGAPI pictogram exhibited a predominantly green profile, largely attributable to the absence of complex extraction or purification processes, the use of a water-based micellar mobile phase, and the removal of volatile organic solvents. The replacement of traditional acetonitrile- or methanol-based mobile phases with an aqueous system incorporating SDS and Brij-35 led to a notable decrease in solvent toxicity, flammability, and atmospheric vapor emissions, concurrently enhancing laboratory safety. Concurrently, the AGREE metric was employed to quantitatively assess the method in relation to the 12 principles of Green Analytical Chemistry, utilizing the standard equal weighting scheme [44]. The AGREE assessment validated the proposed method's positive environmental impact, highlighting its minimal sample handling, lower chemical hazards, and reduced waste per analysis, particularly compared with earlier reported LC methods that relied heavily on organic solvents. The evaluations from ComplexGAPI and AGREE collectively demonstrate that the proposed method is both analytically efficient and environmentally preferable for the routine determination of the studied drugs, compared with previously published methods (Table 5).

**Table 5:** Comparison of the proposed method with some reported methods

	Proposed method	Reported method [25]	Reported method [30]
Technique	HPLC–UV	HPLC–UV	HPLC–UV
Optimization strategy	RSM	OFAT	OFAT
Stationary phase	RP-C18	RP-C18	RP-C18
Organic modifier	Totally free	Acetonitrile: phosphate buffer,@@ pH 6 (45:55, v/v)	Acetonitrile: phosphate buffer, pH 3 (30:70, v/v)
Analytes similarity	TRZ, TAD, and TMS	TAD and TMS	TRZ and TMS
Run time	8 min	4 min	8 min
Complex GAPI assessment			
AGREE assessment			

An analysis of previously documented LC techniques for assessing binary mixtures of the chosen analytes, in comparison with the current method, reveals several significant benefits of the proposed approach. The developed method contrasts with traditional approaches, which predominantly use organic-solvent-containing mobile phases and standard optimization techniques. Instead, it employs an organic-solvent-free mobile phase and has been systematically optimized through a QbD-based strategy. The proposed procedure enables simultaneous determination of TRZ, TAD, and TMS in a single chromatographic run. The findings presented in Table 5 indicate that the developed method exhibits enhanced ecological performance relative to other published techniques, particularly in optimization efficiency, multi-analyte capability, and overall sustainability.

## Conclusion

A novel Quality by Design (QbD)- based mixed-micellar HPLC approach was developed and validated for the simultaneous quantification of terazosin (TRZ), tadalafil (TAD), and tamsulosin (TMS). The proposed method achieved successful chromatographic separation using an environmentally friendly aqueous mobile phase consisting of SDS (150 mM) and Brij-35 (20 mM), formulated in a 10 mM sodium dihydrogen phosphate buffer (pH 4.84), thereby obviating the need for organic modifiers typically employed in conventional liquid chromatography. The application of central composite design facilitated a systematic assessment of essential factors and their interactions, yielding a strong design space that ensured high resolution, precise quantification, and reduced analysis time. The approach exhibited exceptional system suitability, linearity across the examined range, and high sensitivity, supporting its suitability for routine quality control analysis. Moreover, the evaluation of greenness using the ComplexGAPI and AGREE metrics validated the enhanced sustainability profile of the new approach compared with documented LC procedures.

## Supplementary Information

Additional file 1: Supplementary materials.

## Data Availability

All data supporting the findings of this study are available within the paper and its Supplementary Information. Further inquiries can be directed to the corresponding authors.
