# Supplementary material for: A QbD-based method for the simultaneous determination of tadalafil, terazosin, and tamsulosin using organic-solvent free mixed-micellar HPLC: a sustainable green approach
Source: BMC Chem. 2026 Jul 1;20(1):144. doi: 10.1186/s13065-026-01866-2 (PMC13330497; doi:10.1186/s13065-026-01866-2)
Supplement: Supplementary file 1 — Additional file 1: Supplementary materials. [file 13065_2026_1866_MOESM1_ESM.pdf]

A QbD-Based Method for the Simultaneous Determination of Tadalafil, Terazosin, and Tamsulosin using Organic-Solvent Free Mixed-Micellar HPLC; A Sustainable Green Approach

Yahya Bin Abdullah Alrashdi[1], Galal Magdy[2,3], Mohamed M. Osman[4,5], Samy G. Alamir[4,6], Sami El Deeb[7*], Ahmed Al-Harrasi[4], Adel Ehab Ibrahim[8,4*]

1 College of Health Sciences, University of Nizwa, Nizwa 616, Oman; alrashdiyahya@unizwa.edu.om (Y.A)

2 Pharmaceutical Analytical Chemistry Department, Faculty of Pharmacy, Kafr-Elsheikh University, Kafr Elsheikh 33511, Egypt; galal_magdy@pharm.kfs.edu.eg (G.M)

3 Department of Pharmaceutical Analytical Chemistry, Faculty of Pharmacy, Mansoura National University, Gamasa, 7731168, Egypt.

4 Natural and Medical Sciences Research Center, University of Nizwa, Nizwa 616, Oman; adel@unizwa.edu.om (A.I), aharrasi@unizwa.edu.om (A.A); mohamed.osman@unizwa.edu.om (M.O)

5 Department of Pharmaceutical Analytical Chemistry, Faculty of Pharmacy, Mansoura University, Mansoura 35516, Egypt, mohamed.osman@mans.edu.eg (M.O)

6 Pharmaceutical Analytical Chemistry Department, Faculty of Pharmacy, Ain Shams University, Organization of African Unity Street, Abassia, 11566, Cairo, Egypt; sami.goerge@pharma.asu.edu.eg (S.A)

7 Institute of Medicinal and Pharmaceutical Chemistry, Technische Universitaet Braunschweig, 38106 Braunschweig, Germany; s.eldeeb@tu-bs.de

8 Department of Pharmaceutical Analytical Chemistry, Faculty of Pharmacy, Port-Said University, Port Said, 42511, Egypt.

* Correspondence: adel@unizwa.edu.om; s.eldeeb@tu-bs.de

Supplementary materials table S1: Central composite design for three independent variables

Standard	Run	Factor A	Factor B	Factor C
		pH	Brij Conc. (mM)	SDS Conc. (mM)
11	11	4.5	9.5	100.0
1	12	3.5	16.0	70.0
2	5	5.5	16.0	70.0
5	13	3.5	16.0	130.0
6	7	5.5	16.0	130.0
13	8	4.5	25.5	49.5
9	1	2.8	25.5	100.0
18	2	4.5	25.5	100.0
15	4	4.5	25.5	100.0
16	9	4.5	25.5	100.0
19	15	4.5	25.5	100.0
17	19	4.5	25.5	100.0
10	3	6.2	25.5	100.0
14	14	4.5	25.5	150.5
3	10	3.5	35.0	70.0
4	17	5.5	35.0	70.0
7	18	3.5	35.0	130.0
8	16	5.5	35.0	130.0
12	6	4.5	41.5	100.0

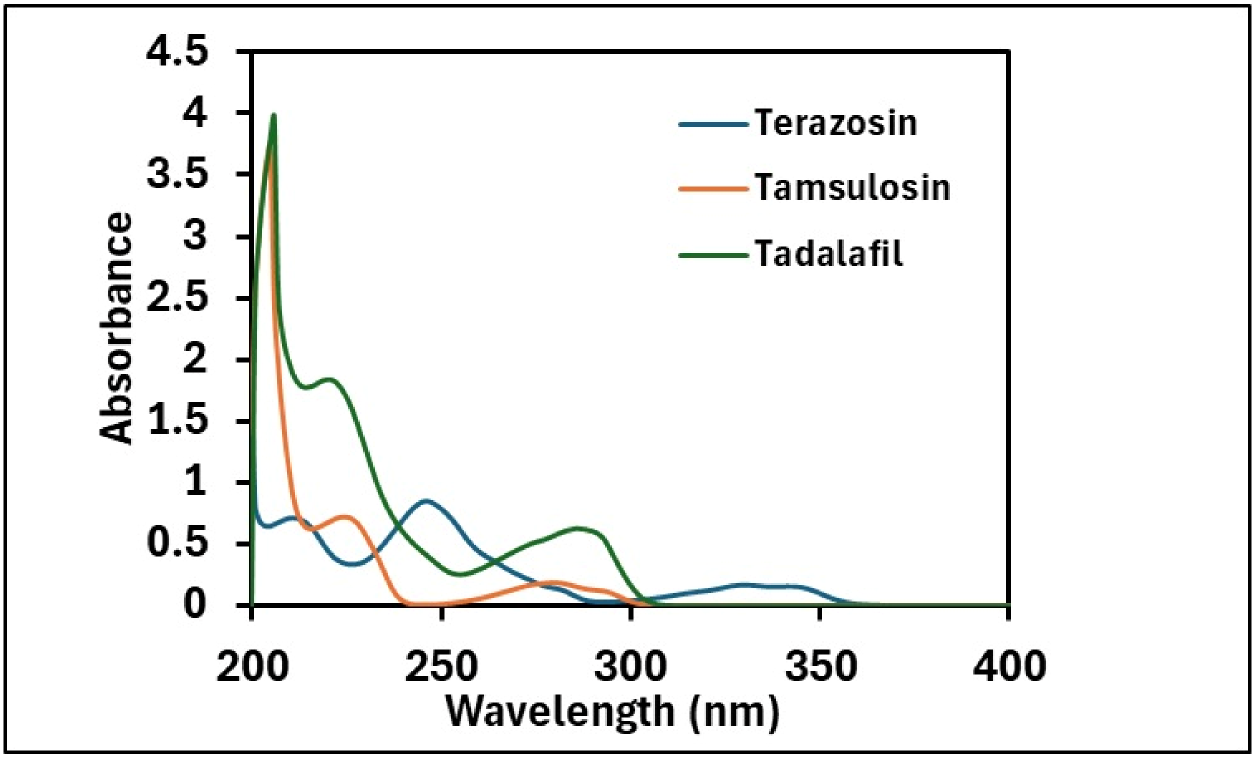

Supplementary materials Figure S2: Overlaid UV/VIS spectra of the drugs under study

Supplementary materials Table S3: Chromatographic factors and their coded and actual levels used in the Central Composite Design (CCD)

Factor	Axial Low ($-\alpha$)	Low (−1)	Center (0)	High (+1)	Axial High ($+\alpha$)
Factor A<br>pH	2.8	3.5	4.5	5.5	6.2
Factor B<br>Brij concentration (mM)	9.5	16.0	25.5	35.0	41.5
Factor C<br>SDS concentration (mM)	49.5	70.0	100.0	130.0	150.5

Supplementary materials Table S4: Robustness results for the assay of the drugs under the proposed method.

Analyte	Flow rate (± 0.1 mL/min)*	Column temperature (± 2°C)*	Wavelength (± 2 nm)*
TRZ	100.07 ± 0.67	98.60 ± 1.97	99.26 ± 1.53
TAD	99.57 ± 2.94	99.62 ± 1.00	100.93 ± 1.49
TMS	101.95 ± 1.69	100.65 ± 0.58	98.76 ± 1.61

* The data represents the average recovery results obtained for each cited drug's concentration after +/- changes in the corresponding chromatographic condition (% recovery ± RSD).
